# Field Validation of Concrete Transport Property Measurement Methods

**DOI:** 10.3390/ma13051166

**Published:** 2020-03-05

**Authors:** Ahmed Abd El Fattah, Ibrahim Al-Duais, Kyle Riding, Michael Thomas, Salah Al-Dulaijan, Mesfer Al-Zahrani

**Affiliations:** 1 Architecture Department, King Fahd University of Petroleum and Minerals, PO. Box 215, Dhahran 31261, Saudi Arabia; 2 Civil Engineering Department, King Fahd University of Petroleum and Minerals, Dhahran 31261, Saudi Arabia; g200927910@kfupm.edu.sa (I.A.-D.); sud@kfupm.edu.sa (S.A.-D.); mesferma@kfupm.edu.sa (M.A.-Z.); 3 Civil Engineering Department, University of Florida, Gainesville, FL 32611, USA; kyle.riding@essie.ufl.edu; 4 Civil Engineering Department, University of New Brunswick, Fredericton, NB E3B 5A3, Canada; mdat@unb.ca

**Keywords:** binding capacity, corrosion, chloride diffusion, durability, formation factor, void ratio, marine exposure site

## Abstract

Reinforcing steel corrosion, caused by chloride ingress into concrete, is the leading cause of reinforced concrete deterioration. One of the main findings in the literature for reducing chloride ingress is the improvement of the durability characteristics of concrete by the addition of supplementary cementitious materials (SCMs) and/or chemical agents to concrete mixtures. In this study, standard ASTM tests—such as rapid chloride permeability (RCPT), bulk diffusion and sorptivity tests—were used to measure concrete properties such as porosity, sorptivity, salt diffusion, and permeability. Eight different mixtures, prepared with different SCMs and corrosion inhibitors, were tested. Apparent and effective chloride diffusion coefficients were calculated using bound chloride isotherms and time-dependent decrease in diffusion. Diffusion coefficients decreased with time, especially with the addition of SCMs and corrosion inhibitors. The apparent diffusion coefficient calculated using the error function was slightly lower than the effective diffusion coefficient; however, there was a linear trend between the two. The formation factor was found to correlate with the effective diffusion coefficient. The results of the laboratory tests were compared and benchmarked to their counterparts in the marine exposure site in the Arabian Gulf in order to identify laboratory key tests to predict concrete durability. The overall performance of concrete containing SCMs, especially fly ash, were the best among the other mixtures in the laboratory and the field.

## 1. Introduction

Corrosion of reinforcing steel is the most common cause of reinforced concrete deterioration [1,2]. While the high pH environment of concrete normally protects reinforcing steel from corrosion, presence of chlorides at high rates in pore solutions, which reduces alkalinity, or at the steel–concrete interface can initiate corrosion. The chloride concentration needed to disrupt this protection is called the chloride threshold [3]. Chloride ingress typically follows three forms according to [4,5] that include: ingress by diffusion, ingress by capillary suction, and ingress by absorption and permeation. Diffusion is described as the movement of chloride (or other water-soluble ions) under a chemical differential. In marine environments, concrete is subjected to chloride diffusion from the sea water. The rate of diffusion is influenced by the diffusion coefficient as described by Fick’s second law of diffusion [6,7,8] as shown in Equation (1).

(1) $$\frac{\partial }{\partial x}\left(D_{c\;}\frac{\partial C}{\partial x}\right)+\frac{\partial }{\partial y}\left(D_{c\;}\frac{\partial C}{\partial y}\right)\;+\frac{\partial }{\partial z}\left(D_{c\;}\frac{\partial C}{\partial z}\right)=\;\frac{\partial C}{\partial t}\;$$

where *D_c_* is the diffusion coefficient (m^2^/s), *C* is the chloride concentration (%), and *t* is time (*s*). Concrete service life is often predicted by modeling the chloride ingress into the concrete. Once the chloride content at the depth of the reinforcing steel has been reached, corrosion is initiated in the model.

The diffusion coefficient in Equation (1) accounts for the rate at which chloride ions can diffuse through the concrete pores. The concrete diffusion coefficient is a function of the chloride diffusion in pore solution with infinite dilution, the total porosity, and the porosity connectivity and tortuosity. The effective diffusion coefficient is the diffusion coefficient separated from the effects of chloride binding. Due to alumina hydration products in the concrete, part of the diffused chloride is bound by the formation of complex salts such as Friedel’s salt or Kuzel’s salt. Some portion of the chlorides are also bound through physical adsorption on C-S-H [9]. Chloride binding can slowly ingress into concrete. An apparent diffusion coefficient is often used that combines the effects of diffusion and chloride binding into one term. The apparent diffusion coefficient can be easily fit to experimental data obtained from bulk concrete diffusion tests such as ASTM C1556 without having to measure the chloride binding or separate out the two effects.

While the literature is full of data on the concrete apparent diffusion coefficient for different mixtures, there is scant data on the effective chloride diffusion coefficient in concrete after chloride binding has been separated [10,11].

One of the best ways to protect concrete reinforcing steel from corrosion and increase the service life is to reduce the concrete porosity and permeability [4,12,13]. Concrete permeability and diffusivity can be reduced by the use of supplementary cementitious materials (SCMs) such as slag cement, fly ash, or silica fume or use of a lower water-to-cementitious materials ratio (w/c) [14,15,16,17]. Using of SCMs along with Portland cement causes the hydration of the Portland cement and reaction of the SCMs occur simultaneously and the fines of SCMs particles modify and enhance the microstructure of concrete and reduce its permeability. Chloride diffusion and the influence of adding SCMs and additives while varying the water-to-cementitious material ratio (w/c) is a major study subject in contemporary literature [18,19,20,21,22,23,24,25,26,27,28,29,30,31,32]. For example, [18,19] studied the effect of adding varying amounts of fly ash on the diffusion and reported a decrease in the diffusion coefficient by 70% with 33% fly ash content. In addition, [25] reported an overall improvement as well through the addition of SCMs (silica fume, fly ash, and slag cement) while claiming that the largest improvement was achieved by addition of slag cement (50%) which is in agreement with [27] where 40% slag cement was used. The addition of SCMs is reported to positively influence the binding capacity of concretes made with such materials [16,18,19,28,33,34,35]. Corrosion inhibitors are chemical compounds added to reinforced concrete to reduce steel corrosion [36] by reacting with an anodic half-cell, such as calcium nitrite corrosion inhibitor (CNI), cathodic half-cell, or both such as is the case with migrating corrosion inhibitor (MCI). CNI is a calcium nitrite-based admixture that oxides the steel to form ferric oxide to resist chloride attack. The CNI decreases chloride binding capacity due to the adsorption of nitrites on cement [37], however, it performs the best when combined with cementitious materials [38]. MCI, migration corrosion inhibitor, migrates in concrete towards reinforcement and forms a protective monomolecular layer. MCI can slowly migrate in the concrete, especially when the concrete has a dense microstructure, and delay the protection [39]. Caltite is a soluable ammonium stearate that contains asphalt particles. It reacts with calcium hydroxide to produce a water-repellent membrane in the pores and reverse the capillary suction, whereas the asphalt particles block capillaries upon moisture ingression [40]. Caltite performs the best when the concrete is exposed to hydrostatic pressure [41].

Many test methods to measure the concrete resistance to chloride ingress and potential durability have been developed, all of which attempt to measure properties governed by the concrete pore structure. Recent work on concrete electrical properties has shown promise in its ability to characterize the concrete pore structure by normalizing for the pore solution conductivity to get the formation factor. Formation factor *F* can be calculated using Equation (2)

(2) $$F=\frac{\rho_{0}}{\rho }=\frac{D}{D_{0}}.\;$$

where *ρ*_0_ is the concrete resistivity (Ω·m), *ρ* is the pore solution resistivity (Ω·m), *D* is the chloride diffusion constant in an infinitely dilute solution taken to be 2.032 × 10^−9^ for Cl¯ at 25 °C, and *D*_0_ is the concrete effective diffusion coefficient [42]. The concrete resistivity can be obtained from a number of concrete electrical tests such as ASTM C1202 [43], “Standard Test Method for Electrical Indication of Concrete’s Ability to Resist Chloride Ion Penetration”; ASTM C1760 [44], “Standard Test Method for Bulk Electrical Conductivity of Hardened Concrete”; and AASHTO T358 [45], “Standard Method of Test for Surface Resistivity Indication of Concrete’s Ability to Resist Chloride Ion Penetration”. Formation factor is related to the concrete diffusion coefficient, as shown in Equation (2), and it can be shown that there exists a mathematical relationship between formation factor and water absorption and water permeability [24,46,47,48]. The concrete pore solution resistivity can be obtained from several methods, including pore solution extraction, calculators that use material composition and assumptions about alkali binding and degree of hydration, and sensors (ASTM C1876) [49]. Recent work has suggested that a combination of formation factor, chloride binding coefficients, and empirical coefficients for the type of binding isotherm used could be combined into an equation to calculate the apparent diffusion coefficient [50].

This study examines on the ability of concrete electrical test methods to quantify concrete transport properties, with special focus on the utility of the formation factor. Formation factor was compared to concrete absorption and effective diffusion coefficient for concrete tested in the laboratory. Laboratory-measured values were also compared to performance of concrete samples from the same mixtures exposed to seawater at an outdoor field exposure site [51] to validate the test method performance, especially the formation factor, to predict concrete durability.

## 2. Materials and Mixture Proportioning

Table 1 shows the chemical composition and physical properties of cementitious materials used in this study. Eight different mixtures were prepared as illustrated in Table 2 while maintaining a constant w/c of 0.4. Different supplementary cementitious materials and corrosion inhibitors were used in the study to compare their performance in corrosion. Class F fly ash (FA), slag cement (SC), and silica fume (SF) were used at replacement percentages of 25%, 70%, and 6% by weight of cement, respectively. The mixtures’ ratios and ingredients were identical to the mixtures placed in the exposure site [51], for the validation and comparisons. These ratios also were chosen based on the optimal percentages found in the literature. Migrating corrosion inhibitor (MCI), calcium nitrite corrosion inhibitor (CNI), and Caltite were added to the last three mixes with optimal ratios recommended by the manufacturers, respectively. A high-range water reducing admixture was used in all mixtures. Mixes 1, 2, 6, 7, and 8 contained MIRA110 (5 L/m^3^), whereas mixes 3, 4, and 5 contained WRDA8 (2 L/m^3^) and Viscocrete-SM4110 (1 L/m^3^) (1.6 L/m^3^ for Mix 3).

## 3. Experimental Program and Testing

**Figure 1:**
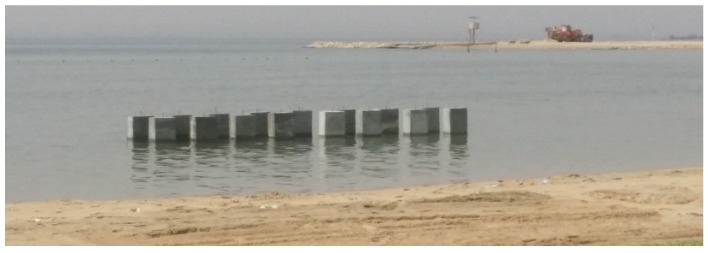
Exposure site.

All concrete mixtures were made by a local ready-mix company and cured after demolding in water basins maintained at 25 °C for 28 days. Nine (100 × 200 mm) and fourteen (75 × 150 mm) cylindrical specimens were made for each mixture and three (150 × 300 mm) concrete cylinders were made from each mixture for compressive strength measurements according to ASTM C39 [52]. In addition, (75 × 150 mm) cylindrical specimen of paste were made for each mixture. Concrete slump and compressive strength were measured, for quality control, and illustrated in Table 3. Fresh concrete temperatures ranged from 26 to 29 °C. Reinforced concrete blocks (23 × 46 × 120 cm) were made from each mixtures, and they have been placed on a marine-exposure site (Figure 1). Each block contained four black steel rebars located at different cover depths; 12.7, 25.4, 38.1, and 50.8 mm. Two biannual tests—chloride profiling and corrosion activity rates, and linear polarization—evaluated the performance of the different mixtures. More information about specimens’ configuration, exposure site setup, and tests results can be found at [51].

The following sub sections discuss comprehensive tests conducted in the laboratory. It should be noted that all tests were performed or started directly after curing the concrete in water for 28 days.

### 3.1. Rapid Chloride Permeability Test

Concrete cylinders with dimensions of 100 × 200 mm were sliced with a rotary saw to make 50 mm thick discs after curing in accordance to ASTM C1202 [43]. Then, they were coated on the sides, excluding the top and bottom of the samples, with epoxy sealant and allowed to cure for one day. After vacuum saturation, the samples were placed into the RCPT cells. The charge passed through the concrete under 60*V* was recorded every half hour for 6 hours for each specimen.

### 3.2. Density, Absorption, and Void Content

Concrete cylinders with the dimensions of 100 × 200 mm were cut into 50 mm thick discs and tested according to ASTM C 642 standard [53]. The samples were oven dried at 110 °C for 24 h and weighed. Then, they were submerged in water and weighed daily until no significant change in the weight was observed. They were placed in boiling water for 5 h and then allowed to cool naturally for 20 h and their mass was recorded. Finally, the specimens were transported to water tank where they were suspended and weighed. The recorded masses where used to calculate the density, volume of permeable voids, and total absorption.

### 3.3. Sorptivity Test

**Figure 2:**
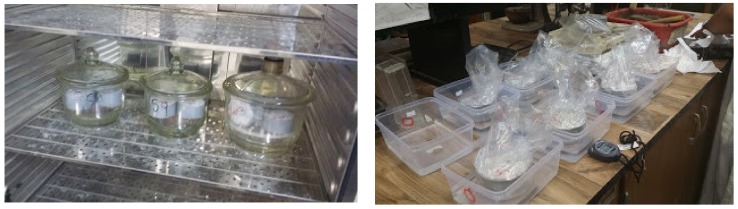
Sorptivity conditioning in KBr (left) and sorptivity testing setup (right).

Concrete cylinders with the dimensions of 100 (diameter) × 50 mm (thickness) were cut following the standard procedure described in the ASTMC1585 [54]. Then they were conditioned in a desiccator that was kept at 50 °C in an oven and a relative humidity of 80% using a saturated solution of potassium bromide (KBr) for three days. Then, they were moved to separate sealable containers for 15 days while ensuring that none of the faces were in contact with the container walls to allow free air flow and then were weighed. Next, the side was coated with epoxy and then one of the faces was covered using a plastic bag and rubber bands or adhesive tape. Then they were suspended with the exposed face downward and water was added up to cover 5 mm of the sides (Figure 2, Right). Finally, the mass was recorded at different times for 9 days as shown in Table 4.

The absorption (*I*) in millimeters was calculated at each time interval. The initial and secondary absorption rates were calculated for each sample using linear regression by the method of the sum of least squares. Moreover, the absorption versus the square root of time was plotted for every mixture. The slope of the first 6 hours represents the initial rate of absorption and the slope from 1 to 9 days represents the secondary rate, as shown in Equation (3)

(3) $$I\;=\;\frac{\Delta m_{t}}{A\times \rho }.\;$$

where Δ*m_t_* is the change in mass at time interval *t* and *A* is the exposed surface area that is in contact with water while *ρ* is taken as the density of water.

### 3.4. Chloride Binding Isotherms

**Figure 3:**
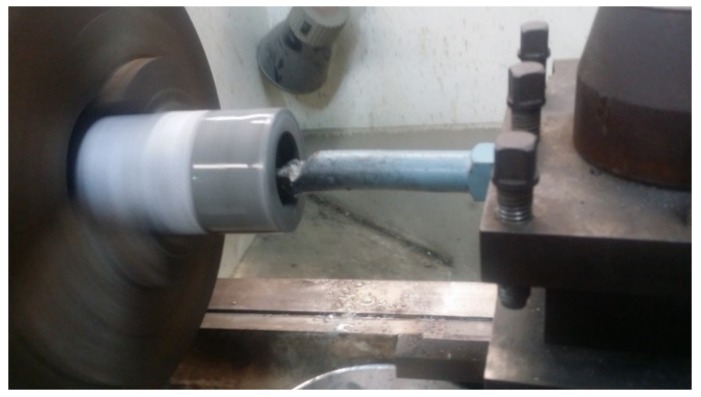
Wet drilling the binding samples using a lathing machine.

This test was conducted following the procedure suggested by [55]. First, cylindrical paste samples were made using a 0.4 w/c and deionized water to eliminate any disturbance in the results caused by the chlorides in the mixing water. Then, the samples were cured in limewater for 14 days in de-aerated containers to avoid any carbonation, and then the central portion of each sample was wet-crushed using a lathe machine as shown in Figure 3. The crushed materials were sieved to pass through a no. 100 sieve and dried in a desiccator filled with activated silica gel. After drying, the samples were placed in a desiccator that had a relative humidity content of 11% at room temperature using a saturated solution of lithium chloride. Finally, samples were divided into 25 g, and exposed to different NaCl concentrations of 0.1, 0.3, 0.5, 1, and 4.2 Molar. After 14 days in the Cl solution, the samples were filtered. The final chloride concentration of the soak solution was measured using potentiometric titration. As illustrated in equation 4, and the binding capacity was calculated based on the difference in the chloride concentration before and after exposure.

(4) $$C_{b}=\frac{35.45\;V\left(c_{1}-c_{o}\right)}{W\times 1000}$$

where *C_b_* is the bound chloride of each mixture in milligram of chloride per gram of paste, *V* is the volume of the salt solution added to each mixture in liters, *W* is the dry weight of the paste used in each exposure cycle in grams, *c_O_* and *c_1_* are the concentration of the salt solution in Molars before and after the exposure respectively.

### 3.5. Bulk Diffusion

**Figure 4:**
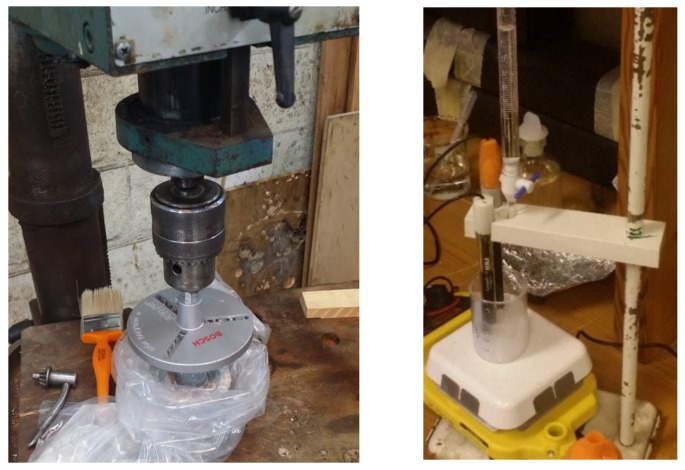
Profile grinding setup (left) potentiometric titration (right).

Concrete cylinders with the dimensions of 75 × 75 mm were coated with epoxy sealant from all sides except the top according to (ASTM C1556 [56]. ASTM C1152 [57]) to direct diffusion of chloride from top only. A minimum of 20 mm from the bottom of one sample was sliced to determine the initial chloride content. Then, the coated specimens were immersed into a saturated calcium hydroxide solution for a minimum of 24 h period and weighed until no significant change in weight was recorded. Then the specimens were exposed to sodium chloride solution for 35 and 183 days. Finally, they were ground by a bench drill press and a diamond coring drill bit (Figure 4). Each specimen was cleaned before grinding of next layer to avoid contamination. The powder from each layer was collected and pulverized to pass through no. 100 standard sieve then dissolved in nitric acid and left to be digested for at least 24 h. After digestion, the remaining solution was filtered and analyzed using potentiometric titration against silver nitrate (AgNO_3_) as described in standard (ASTM C1556) [56].

The concrete apparent chloride diffusion coefficient was calculated assuming a constant diffusion coefficient with time using Fick’s law of diffusion and the error function as shown in Equation (5) was fitted to the experimental profile using least squares to find *C_s_ and D_a_*:

(5) $$C\left(x,t\right)=Cs-\left(Cs-Ci\right)\times \mathrm{erf}\left(\frac{x}{\sqrt{4\times Da\times t}}\right)$$

where *C(x,t)* is the chloride concentration at different depth *x*(m) and time *t(s),* and it is measured as percent weight of concrete, *C_s_* is the chloride concentration on the surface of the concrete at the interface between the salt solution in the ponding container and concrete, *C_i_* is the initial chloride concentration before ponding, *D_a_* is the bulk diffusion coefficient (m^2^/s), and *erf* is the Gauss error function described by Equation (6)

(6) $$\mathrm{erf}\left(z\right)=2/\pi \times \int_{0}^{z}\exp\left(-u^{2}\right)du$$

### 3.6. Effective Chloride Diffusion Coefficient

Effective concrete chloride diffusion coefficient at 28 days was calculated for each mixture using the measured chloride profiles ponding samples, the chloride binding results, the volume of permeable voids, and concrete density. The chloride ingress was modeled using an explicit finite difference-based approach to approximate the chloride diffusion. The chloride binding was taken into account at each time step by calculating an apparent diffusion coefficient in Equation (7) [58] using the Freundlich isotherm chloride binding coefficients calibrated to the experimental chloride binding results as described in Equation (8).

(7) $$D_{c}^{*}=\frac{D_{c}}{1+\frac{1}{\omega_{e}}\alpha \beta C_{fc}^{\beta -1}}$$

(8) $$C_{bc}=\alpha C_{fc}^{\beta }$$

where D_c_^*^ is the apparent diffusion coefficient for that time step and node (m^2^/s), *D_c_* is the concrete effective diffusion coefficient at the time step (m^2^/s), *ω_e_* is the volume of permeable voids, *C_bc_* is the bound chloride content, *C_fc_* is the free chloride concentration (kg/m^3^), and *α* and *β* are fitting coefficients to the chloride binding data *C_bc_* and *C_fc_*.

*D_c_* is known to change with time as shown in Equations (9) and (10) [10]

(9) $$D_{c}=D_{28}{\left(\frac{28}{t}\right)}^{m}+D_{28}{\left(\frac{28}{36,500}\right)}^{m}\left(1-{\left(\frac{28}{t}\right)}^{m}\right)$$

(10) $$m=0.26+0.4\left(\frac{FA}{50}+\frac{SG}{70}\right)$$

where *D_28_* is the concrete effective diffusion coefficient at 28 days, *t* is the concete age (days), FA and SG are the fly ash and slag cement content as a mass percentage of the cementitious materials used. 

## 4. Results and Discussion

### 4.1. Rapid Chloride Permeability Test

Rapid chloride permeability results are shown in Table 5. The concrete formation factor was calculated from RCPT data collected during the first hour of the test and the pore solution conductivity was calculated using the NIST pore solution conductivity calculator (http://ciks.cbt.nist.gov/poresolncalc.html) and cementitious material composition shown in Table 1 for all mixtures except those with corrosion inhibitors or hydrophobic admixtures. Table 5 shows the calculated concrete formation factors. FA and SC mixtures had the lowest value of electrical permeability and were classified in accordance to the ASTM standard as having low permeability. This was not surprising since they both contained significant quantities of SCMs. The SF mixture was lower than the control Portland cement sample, but was higher than FA and SC mixtures. This illustrates the benefits of using formation factor to correct for the lower pore solution conductivity found with silica fume. On the other hand, Caltite, CNI, and type V concretes showed the highest values and exceeded the standard mixture values by 8.8%, 27%, 48% respectively. It should be noted that CNI significantly increases the conductivity of the pore solution and, hence, the RCPT value does not mean that the chloride permeability was increased [45]. The test was mainly designed as indication of the susceptibility of concretes to allow chloride to pass depending on their microstructure.

### 4.2. Density, Absorption, and Void Ratio

As shown in Table 6, two samples were tested for each mixture. With void value of 9.02%, SC exhibited the lowest void ratio. This was attributed to the relatively high dense microstructure formed from the high percentage of slag. FA recorded the least absorption rate of 5.25%. All other mixtures exhibited lower values than the standard mixture (13.9% void, 6.44% absorption) with SC, CNI, and FA mixtures showing the lowest values. The results do not match up with that seen for RCPT and formation factor. This is likely because connectivity of pores is just as important as total content of pores, contributing to the low correlation seen between these parameters [59].

In terms of density, it was shown that the CNI mixture exhibited the highest bulk and dry densities at values of 2.33 and 2.21 g/m^3^.

### 4.3. Sorptivity

**Figure 5:**
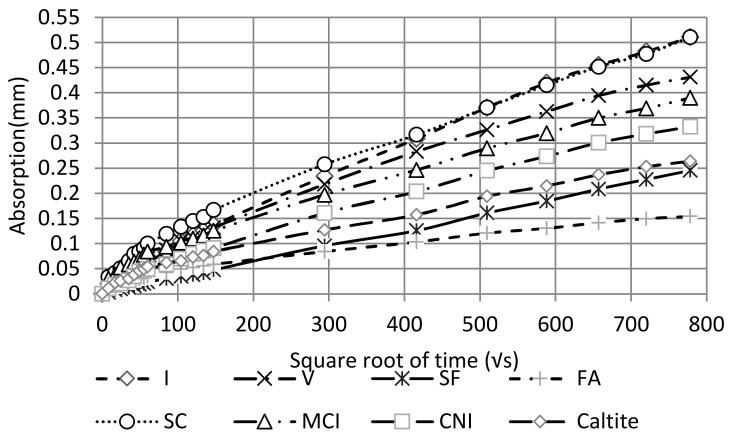
Sorptivity curves.

Figure 5 shows the concrete water sorptivity results. The sorptivity was shown to be highest with the SC mixture. On the other hand, SF and FA mixtures showed the lowest overall absorption during the seven-day testing period. This result is in agreement with the literature [60,61]. It is worth noting that all mixtures had a lower overall absorption than the standard mixture except for slag cement which exhibited the highest initial absorption. This might be attributed to the high interaction between slag and water that needed more time for hydration, especially when the slag percentage exceeded 40% [14,62].

**Figure 6:**
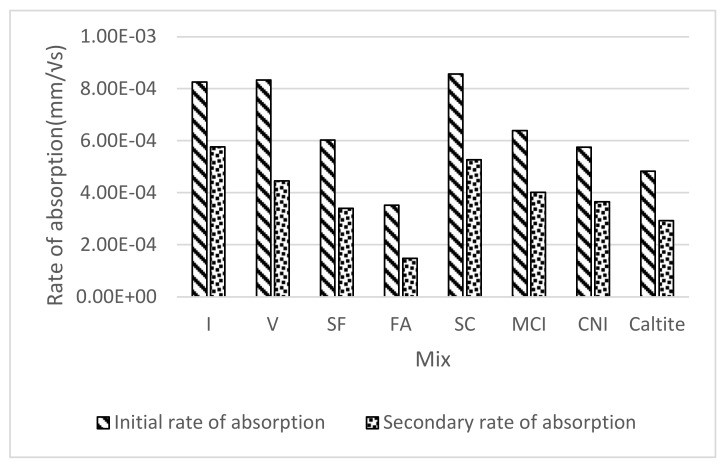
Comparison of the different rates of absorption.

As illustrated in Figure 6, the rate of absorption was observed to decrease significantly with time which indicated that the role of sorptivity in chloride transportation into concrete might be limited to early exposure times. Moreover, the SC mixture showed the highest primary rate of absorption and could help explain the higher-than-expected chloride concentrations in the bulk diffusion testing at 35 days of exposure (Section 4.5). On the other hand, the FA and SC mixtures exhibited the lowest total absorption and rates of absorption that indicated an enhanced microstructure and more resistivity to chloride diffusion.

**Figure 7:**
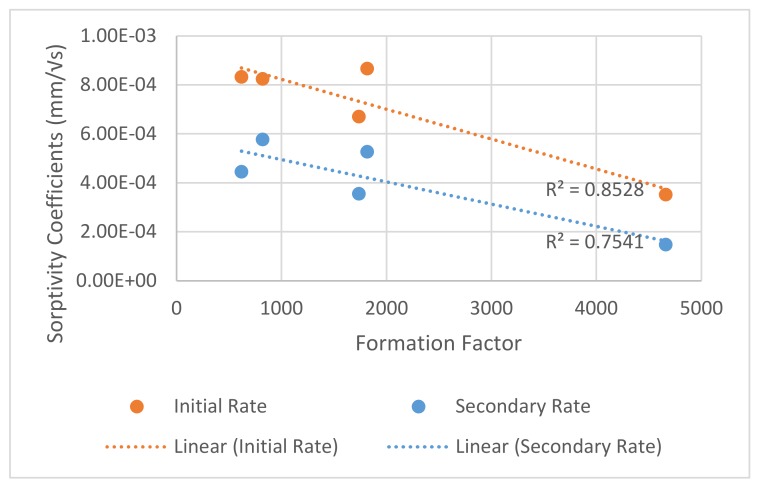
Comparison of formation factor and sorptivitiy coefficients.

A good linear correlation, with average R^2^ value of 0.8, was found between the formation factor and sorptivitity coefficients as shown in Figure 7. This was likely because the sorptivity was so dependent on the pore volume and connectivity [63], as was the formation factor. The mixture that fell below both trend-lines was the silica fume mixture, indicating that potentially the pore solution conductivity calculated by the NIST calculator could be showing a higher pore solution conductivity than the actual concrete mixture. This could be because of leaching that occurred during curing.

### 4.4. Chloride Binding Isotherms

**Figure 8:**
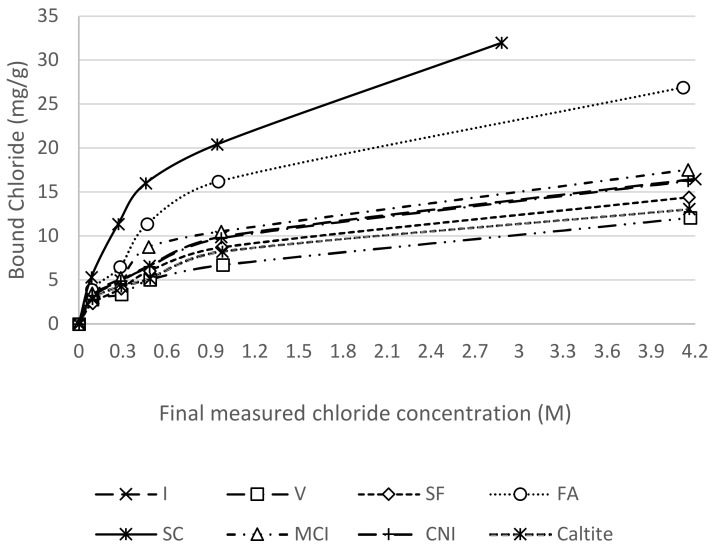
Binding at different concentrations and their logarithmic models.

As illustrated in Figure 8, SC exhibited the highest binding capacity with increasing concentrations of NaCl in the environment. FA also improved significantly the chloride binding. Type V cement, as expected, had the lowest binding capacity due to the lower amount of alumina in the cement compared to Type I cement. Table 7 shows the calibrated binding coefficients using Equation (8). The FA and SC mixtures showed the highest levels of bound chlorides, owing mostly to the high alumina content [64]. SF bound less chloride compared to Mixture I due to the dilution of C_3_A and the reduction of pH level that caused release of chloride [65]. CNI recorded less binding capacity than mixture I, confirming the findings in the literature [37].

### 4.5. Bulk Diffusion

**Figure 9:**
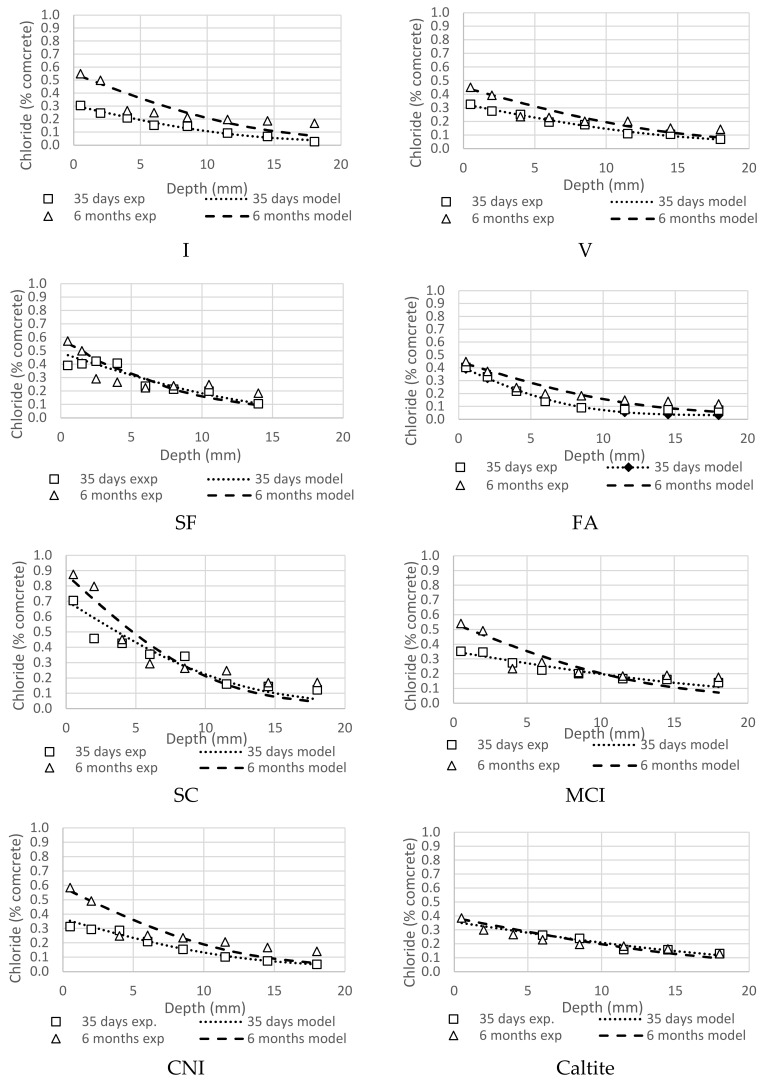
Comparison of one month and six months of ponding for all mixtures.

SC illustrated the highest overall chloride profile along its depth during the first 35 days of ponding (Figure 9). SC had the highest chloride surface concentration owing to its high binding capacity and high absorption rate (Figure 8). However, it showed little increase in the six-month ponding results except near the surface as shown in Figure 9, and can be explained by its ability to reduce permeability with time [66]. The FA mixture had the least chloride concentration at different depths, which agreed with absorption and void ratio experiments. This indicated that FA mixture had very dense microstructure which helped in hindering the movement of chlorides in the matrix. The chloride diffusion depth was least at SF mixture which showed a maximum of 13 mm penetration. This also implied denser microstructure at higher depths. The corrosion inhibitors had little effect as expected on the chloride migration. Caltite had some benefits on chloride concentration with age.

On the other hand, the chemical additives seemed to have a minimal overall effect on the chloride profile during the first 35 days of ponding. However, their effect was more pronounced after the six-month ponding with great improvement in the profile, especially the caltite then MCI [13].

The effect of the addition of SCMs and chemical additives seemed to be more significant with the passage of time as illustrated by the six-month ponding results.

### 4.6. Effective Chloride Diffusion Coefficient

**Figure 10:**
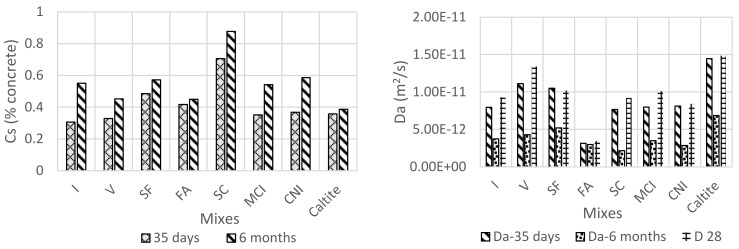
Surface chloride (Left) and diffusion coefficient (Right).

Figure 10 shows the calculated chloride surface concentrations, *C_s_*, diffusion coefficients, *D_a_*, and *D_28_*, for the concrete bulk diffusion experiments. SC mixture showed the highest decrease, after six months, in the diffusion coefficient which indicated a much slower transfer of chlorides into the concrete. Moreover, the addition of the chemical additives seemed to have a significant effect on the diffusion coefficient which decreased by 55%, 54%, and 73% in the cases of MCI, CNI, and caltite mixtures respectively. In terms of the surface concentration, SC mixture was shown to have the highest surface concentration which could be attributed to its considerably higher binding capacity and initial rate of absorption, while type V concrete exhibited the lowest in the six-month ponding. The lower capacity of V mixture to bind chlorides indicated that the penetration of chlorides might be easier in this mixture, which in turn can be detrimental to its durability in longer exposure periods.

Considering the expected level of error in chloride profile and binding measurements, the maximum 23% difference between *D_a_* and *D_28_* was small. The higher values on average of the effective diffusion coefficient was most likely because it was a 28-day value instead of an average of the diffusion coefficient between 28 and 63 days and chloride binding was explicitly accounted for. Use of the simplified Equation (5) to calculate the concrete diffusion coefficient from 35-day ponding tests for service life modeling may give similar results. While large electrical potentials have been shown to cause microstructural changes to the concrete and the proportion of large harmful pores [67], it is not believed that this factor caused a significant increase in the formation factor measured. The formation factor was calculated from values during the early stages of the ASTM C1202 test [43], limiting the time at which the high electrical potential could have affected the concrete microstructure and altered the calculated formation.

Table 8 and Table 9 rank the performance of the mixtures in the laboratory and field, respectively, following pairwise comparison method. Generally, concretes made with cementitious materials outperformed those made with corrosion inhibitors and hydrophobic materials. Laboratory results agreed with field results, as shown in Table 8 and Table 9, that FA and SC mixtures provided the best results for the experimental program, whilst I and V mixtures provided the worst results. It is noteworthy that corrosion inhibitors ingress slowly in the concrete towards the steel, especially in the denser microstructures [13]. This may explain why SCMs performed better. They were better able to keep the chlorides out of the concrete because of their pore system densification.

The highest formation factor was recorded for FA then SC, SF, I, and V (Table 5). This was consistent with the results of RCPT, bulk diffusion and void ratio. As seen in Table 9, the formation factor was correlated to the steel corrosion activities of the mixtures in the exposure site [51]. It also was correlated satisfactorily to the diffusion coefficient readings from the exposure site.

**Figure 11:**
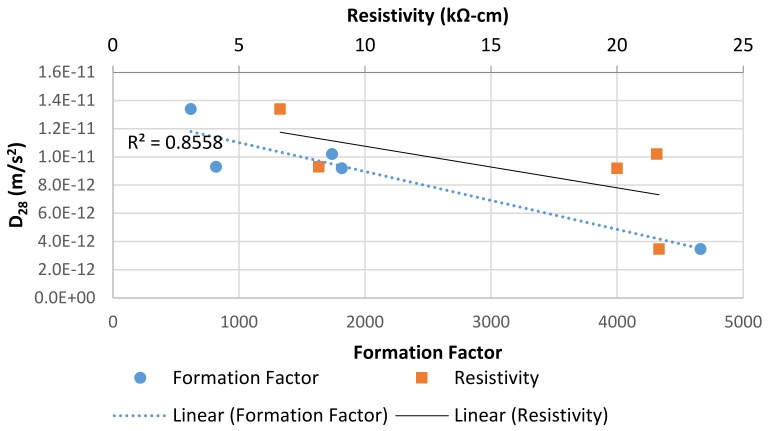
Comparison of effective diffusion coefficient D_28_ to concrete properties measured by electrical tests measured after 28 days of curing.

A comparison of the concrete effective diffusion coefficient, *D_28_*, versus measured concrete electrical properties was performed in order to determine the ability of commonly used concrete quality control methods to predict the concrete durability. Figure 11 showed that the concrete effective diffusion coefficient after 28 days of ponding was correlated to concrete formation factor. The correlation found indicates that the formation factor could be used as a reasonable surrogate for the effective diffusion coefficient in service life modeling and quality control applications.

There was good agreement among the experiments of absorption, void ratio, sorptivity, and bulk diffusion because the void ratio influenced greatly the sorptivity and bulk diffusion. Although SC mixture recorded the lowest void ratio, its absorption and sorptivity was relatively high for the same days of curing. This could be explained by the need of SC mixture to absorb water for more hydration, according to the literature, especially when the slag percentage exceeded 40% [14,62]. Nevertheless, its great binding capacity and its ability to reduce permeability with curing time were seen in the laboratory and field tests. The RCPT test was in agreement with bulk diffusion test by confirming the superiority of the FA and SC mixtures over the other mixtures.

The mixtures were ranked according to their overall performance using pairwise comparison method for all of the tests. There was good agreement between the laboratory and field outcomes. Besides the samples made with corrosion inhibitors, the formation factor was able to predict the relative field performance of the concrete mixtures. The performance of corrosion inhibitors materials, however, was improved in the field which might be attributed to their better performance at larger depth due to decrease of leaching out and better formation and blending. Future work to quantify the formation factor of the mixtures containing corrosion inhibitors through pore solution extraction is needed.

The absorption, void ratio, and the binding capacity tests along with formation factor could be indicative, as short term tests, of the long term performance of the different mixtures since their results predicted the field performance.

## 5. Conclusions

Based on this study, the following conclusions can be drawn:

- Apparent diffusion coefficient calculated from concrete bulk diffusion tests using the error function and the effective diffusion coefficient at 28 days were similar. This implies that the simple approach used with the error function provides a good index of concrete quality.
- A good correlation was found between the concrete formation factor and effective diffusion coefficient as predicted by theory. Because the effective diffusion coefficient is a fit value that involves the use of several other parameters—such as chloride surface concentration, chloride binding isotherm, and concrete chloride profile—some error in the values was expected.
- The laboratory and field results showed that the cementitious materials outperformed the corrosion inhibitors. The use of SCMs provided the largest benefit in reducing chloride diffusion. The corrosion inhibitors showed better performance in the field, especially at larger depths, and after six months of exposure in the laboratory which could be attributed to the effectiveness of corrosion inhibitors at larger depths and leaching out decrease overtime.
- FA and SC mixtures performed the best compared to the other mixtures for the conducted experimental program in the laboratory and in the field, whereas I and V mixtures delivered the least quality performance as expected.
- Formation factor predicted the relative performance of concrete mixture in the field. The formation factor could be used as a reasonable surrogate for the effective diffusion coefficient in service life modeling and concrete durability applications. Since the formation factor can be estimated quickly and at a low cost, it could be used more frequently to measure durability than currently used tests. More work on the effect of curing on formation factor is needed.
- Absorption and binding capacity tests along with the formation factor could be indicative of long-term performance of concrete mixtures since their results agreed with the performance of the concrete in the marine exposure site.

## Figures and Tables

**Table 1:** Chemical and physical properties of cementitious materials.

Chemical and Physical Analysis (%)	OPC	Cement Type V	Silica Fume	Fly Ash	Slag
SiO_2_	20.8	20.97	91	51.47	34.8
Al_2_O_3_	5.37	3.91		24.31	13.4
Fe_2_O_3_	3.32	4.8		8.87	0.62
TiO_2_				1.02	
CaO	63.77	64.27		5.15	43.4
MgO	2.08	1.97		3.50	5.44
SO_3_	2.63	1.86		0.23	0.34
Loss on Ignition (L.O.I)	1.34	2.16	6.00	0.53	
Insoluble residue (I.R.)	0.39	0.60			0.34
Na_2_O_eq_	0.52	0.48			0.56
K_2_O				1.47	
P_2_O_5_				0.257	
C_3_S	53.20	63.84			
C_2_S	19.50	11.96			
C_3_A	8.61	2.24			
C_4_AF	10.10	14.61			
C_3_AF + 2C_3_A	27.33	19.09			
Fineness, Air permeability Test (m^2^/Kg)	323	315			

**Table 2:** Mix proportioning.

Mix	W/C	Cement (Kg/m^3^)	Coarse Aggregate (Kg/m^3^)	Sand (Kg/m^3^)	Water (Kg/m^3^)	Silica Fume (Kg/m^3^)	Fly Ash (Kg/m^3^)	Slag Cement (Kg/m^3^)	Notes
I	0.4	340	*1070*	*775*	136	-	-	-	Type OP/CEM 1
V		340	1070	775	136	-	-	-	Type V/ high sulfate-resistant Portland cement
SF		320	1100	735	136	21	-	-	OP + SF
FA		255	1090	735	136	-	85	-	OP + FA
SC		100	1095	735	136	-	-	240	OP + SC
MCI		340	1070	775	136	-	-	-	OP + MCI at 0.6 L/m^3^ of concrete
CNI		340	1070	775	136	-	-	-	OP + CNI at 20 L/m^3^ of concrete
Caltite		340	1070	775	136	-	-	-	Type I + Caltite at 30 L/m^3^ of concrete

**Table 3:** Fresh concrete properties.

Mix	Slump mm	Strength after 28 days-MPa
Type I	30	28.1
Type V	32	28.6
SF	26	30.3
FA	34	39.1
SC	42	28.0
MCI	29	29.2
CNI	31	32.4
Caltite	34	28.1

**Table 4:** Periods at which the mass is calculated in the sorptivity test.

Time	60 s	5 min	10 min	20 min	30 min	60 min	Every Hour up to 6 h	Once a Day up to 7 days	Day 7 to 9, 1 Measurement
**Tolerance**	2 s	10 s	2 min	2 min	2 min	2 min	5 min	2 h	2 h

**Table 5:** RCPT results.

Mix	RCPT	RCPT Permeability Classification	Resistivity (kΩ-cm)	Formation Factor
I	3226	Moderate	8.17	819
V	4008	High	6.63	618
SF	1522	Low	21.57	1738
FA	1140	Low	21.66	4662
SC	1013	Low	20.4	1816
MCI	2491	Moderate	-	-
CNI	4094	High	-	-
Caltite	3509	Moderate	-	-

**Table 6:** Density results.

Sample	Mass (g)						Absorption (%)	Dry Bulk Dens. (g/g)	Bulk Dens. (g/g)	Apparent Dens. (g/g)	Void %
	OD	B.SSD	Suspended	AVG OD	AVG B.SSD	AVG Susp.					
Letter Des.	-	-	-	A	C	D	(C-A/A)*100	A/(C-D)	C/(C-D)	A/(A-D)	(g2-g1)/g2*100
I-1	722.3	768.2	434.4	700.5	745.6	421.6	6.44	2.16	2.30	2.51	13.92
I-2	678.7	723	408.8								
V-1	714.7	762.6	421	712.1	758.4	421.2	6.49	2.11	2.25	2.45	13.72
V-2	709.5	754.1	421.4								
SF-1	759.2	803	451.9	734.4	775.6	439.6	5.61	2.18	2.31	2.49	12.26
SF-2	738.2	779	434.7								
FA-1	639.2	679	384.2	700.3	737	419.6	5.25	2.21	2.32	2.49	11.58
FA-2	761.4	795.1	455								
SC-1	676.6	731.1	409.6	689.9	746.1	417.8	8.15	2.10	2.27	2.54	9.02
SC-2	703.1	761.1	426.1								
MCI-1	785.9	863	473.1	789.6	852.4	476.2	7.95	2.10	2.27	2.52	16.7
MCI-2	793.2	841.7	479.3								
CNI-1	770.9	811.7	463	740.2	779.9	445.2	5.36	2.21	2.33	2.51	11.86
CNI-2	709.5	748.1	427.3								
Caltite-1	759.2	803	451.9	748.7	791	443.3	5.65	2.15	2.27	2.45	12.17
Caltite-2	738.2	779	434.7								

**Table 7:** Chloride binding coefficients.

Mixture	α (mg Cl/ g paste)	β
I	2.0844	0.4159
V	1.4750	0.4210
SF	1.6984	0.4320
FA	2.9867	0.4455
SC	4.3609	0.4338
MCI	2.5519	0.3891
CNI	2.0412	0.4180
Caltite	1.8011	0.3991

**Table 8:** Ranking of mixtures according to their performance in the conducted experiments.

Test	1	2	3	4	5	6	7	8
RCPT	FA	SC	SF	MCI	I	Caltite	V	CNI
Absorption	FA	SF	CNI	Caltite	I	V	MCI	SC
Void ratio	SC	FA	CNI	Caltite	SF	V	I	MCI
Sorptivity	FA	SF	Caltite	CNI	MCI	I	V	SC
Binding capacity	SC	FA	MCI	CNI	I	SF	Caltite	V
Bulk diffusion	FA	SC	SF	CNI	Caltite	MCI	I	V
Overall performance	FA	SC	SF	CNI	Caltite	MCI	I	V

**Table 9:** Performance of the mixtures in the field.

Rank	1	2	3	4	5	6	7	8
Steel corrosion	FA	SC	Caltite	MCI	CNI	SF	I	V
Diffusion coefficient	FA	CNI	I	SF	SC	Caltite	MCI	V
Overall performance	FA	SC	CNI	Caltite	MCI	SF	I	V
